# The Burden of Neurocysticercosis at a Single New York Hospital

**DOI:** 10.1155/2020/8174240

**Published:** 2020-07-03

**Authors:** Amy Spallone, Luboslav Woroch, Keith Sweeney, Roberta Seidman, Luis A. Marcos

**Affiliations:** ^1^Department of Medicine, Division of Infectious Diseases, Baylor College of Medicine, Houston, TX 77030, USA; ^2^Department of Radiology, Stony Brook University Hospital, Stony Brook, NY 11794, USA; ^3^Department of Pathology, MD Anderson Cancer Center, Houston, TX 77030, USA; ^4^Department of Pathology, Stony Brook University Hospital, Stony Brook, NY 11794, USA; ^5^Department of Medicine, Division of Infectious Diseases, Stony Brook University Hospital, Stony Brook, NY 11794, USA; ^6^Global Health Institute, Stony Brook University, Stony Brook, NY 11794, USA; ^7^Department of Molecular Genetics and Microbiology, Stony Brook University, Stony Brook, NY 11794, USA

## Abstract

Neurocysticercosis (NCC), a disease caused by the larval pork tapeworm *Taenia solium*, has emerged as an important infection in the United States. In this study, we describe the spectrum of NCC infection in eastern Long Island, where there is a growing population of immigrants from endemic countries. A retrospective study was designed to identify patients diagnosed with NCC using ICD-9 and ICD-10 codes in the electronic medical records at Stony Brook University Hospital between 2005 and 2016. We identified 52 patients (56% male, median age: 35 years) diagnosed with NCC in the only tertiary medical center in Suffolk County. Twenty-five cases were reported in the last three years of the study. Forty-eight (94%) patients self-identified as Hispanic or Latino in the electronic medical record. Twenty-two (44%) and 28 (56%) patients had parenchymal and extraparenchymal lesions, respectively. Nineteen (41.3%) patients presented with seizures to the emergency department. Six patients (11.7%) had hydrocephalus, and five of them required frequent hospitalizations and neurosurgical interventions, including permanent ventriculoperitoneal shunts or temporary external ventricular drains. No deaths were reported. The minimum accumulated estimated cost of NCC hospitalizations during the study period for all patients was approximately 1.4 million United States dollars (USD). In conclusion, NCC predominantly affects young, Hispanic immigrants in Eastern Long Island, particularly in zip codes correlating to predominantly Hispanic communities. The number of cases diagnosed increased at an alarming rate during the study period. Our study suggests a growing need for screening high-risk patients and connecting patients to care in hopes of providing early intervention and treatment to avoid potentially detrimental neurological sequelae.

## 1. Introduction

Neurocysticercosis (NCC) is an infection of the central nervous system with the larval pork tapeworm *Taenia solium* [1]. The infection is acquired by the ingestion of an adult tapeworm's ova released in the feces of an infected tapeworm carrier [2, 3]. NCC is one of the most common parasitic infections of the nervous system, and it is the most common cause of acquired, preventable adult-onset epilepsy and neurological disability worldwide [3–6]. This parasitic infection mainly affects disadvantaged people in endemic regions, which include Latin America, sub-Saharan Africa, and Asia, among others. Thus, the World Health Organization considers NCC as a neglected tropical disease (NTD) [3, 7]. NCC can also be present in industrialized countries where those affected are usually immigrants from endemic areas [8–10]. Local outbreaks of NCC in the United States (US) have become an increasing public health concern [11–14]. It has been estimated that between 1,320 and 5,050 new cases of NCC occur every year in the US, mainly in the Hispanic population, and the calculated annual age-adjusted mortality rate for NCC is 0.06 per million US population [9]. NCC carries significant morbidity due to seizures and other neurological complications from the disease, but experts postulate that early access to treatment and healthcare could improve outcomes in these patients [15, 16]. Moreover, the cost of NCC on the US Healthcare system is estimated to be at least one billion dollars during the last decade [17]. The need for surveillance systems or screening programs is needed in communities where the number of Hispanic immigrants from rural communities is large [9]. To the best of our knowledge, there are no screening programs in the US currently and, thus, NCC remains a disease that is diagnosed after neurological complications develop [5, 9, 16–19]. Immigrants from Central and South America represent one of the fastest-growing populations in Suffolk County, New York (NY) [20, 21]. According to the 2015 census, the total population of Suffolk County was over 1.5 million people and 18.6% of its residents were identified as ethnically Hispanic [21]. Ostensibly, Suffolk County could be considered a high-risk epidemiological area for NCC, but no prior studies have reported the number of NCC cases in this region of NY. The objective of this study is to describe the burden of NCC diagnosed at one of the largest tertiary medical centers on Long Island, NY. Additionally, we postulate that zip codes, in eastern Long Island registered in the electronic medical records (EMR) to NCC cases, correlate with specific communities with large numbers of Hispanic residents.

## 2. Materials and Methods

### 2.1. Study Design

This study was conducted as a retrospective chart review to identify and describe cases of NCC diagnosed at Stony Brook University Hospital (SBUH) from 2005 to 2016. SBUH is a six-hundred-bed tertiary medical center located on Long Island in Suffolk County, NY. Due to limitations in extracting data from medical records before 2005, we chose this year as the start date of our study.

### 2.2. Cases Definitions

NCC cases were classified as definitive or probable according to published criteria. Our data collection occurred prior to the revised diagnostic criteria for NCC by Del Brutto et al. [22] and followed the earlier definitions (Table 1) [23]. Definitive NCC is defined as the presence of one absolute criterion, or the presence of two major criteria, one minor criterion, and one epidemiological criterion. Probable NCC is defined as the presence of one major criterion plus two minor criteria; or the presence of one major, one minor, and one epidemiological criterion; or the presence of three minor criteria plus one epidemiological criterion.

### 2.3. Case Criteria

Cases were initially identified using the International Classification of Diseases, 9^th^ revision (ICD-9) and 10^th^ revision (ICD-10), for neurocysticercosis (ICD-9: 123.1; ICD-10: B69.0), cysticercosis (ICD-9: 123.1; ICD-10: B69.9), and taeniasis (ICD-9: 123.3; ICD-10: B68.9) according to the Centers for Medicare and Medicaid Services [24]. Inclusion criteria for the study were met if patients were discharged from SBUH with an ICD-9 or ICD-10 diagnosis code of NCC between January 1, 2005, and December 31, 2016, and met one of the case definitions defined above. Each patient's chart was analyzed by two investigators to confirm the diagnosis of NCC.

### 2.4. Case Data Abstraction

A dataset was built from the following data extracted from patients' charts: date of admission, demographic data, zip code of residence, occupation, health insurance status, travel history, past medical history, history of consuming raw or undercooked pork, household members with a history of *T. solium* infection, presenting symptoms (e.g., headaches, nausea, vomiting, diplopia, fever, seizures, neurological deficits, and intracranial hypertension), absolute eosinophil count, stool ova and parasite results, histopathology, type of neuroimaging and findings, funduscopic exam, NCC serologic testing (e.g., ELISA or enzyme-linked immunoelectrotransfer blot (EITB)), NCC CSF studies (e.g., EITB and ELISA), treatment with antihelmintics and corticosteroids, surgical intervention, resolution of cyst on imaging (spontaneously or after treatment), and deaths attributed to NCC.

### 2.5. Neuroimaging

A separate dataset was completed for each patient, which included the type of imaging (e.g., computed tomography (CT) or magnetic resonance imaging (MRI)), location of the cyst (i.e., the parenchyma, subarachnoid space, sulcus of the convexity, basal cisterns of the subarachnoid space, ventricular system, or spinal cord), and classification of the phase of the cyst (vesicular, colloidal, granular nodular in degeneration, or calcified). Our study incorporated a neuroradiologist (LW) to review imaging from patients in our study and categorized their NCC based on locations of their cysts (parenchymal, intraventricular, and subarachnoid).

### 2.6. Histopathology

Histopathology specimens were sectioned and prepared by a histotechnologist. Sections were taken on a microtome from paraffin-embedded tissue blocks at five-micron intervals and then processed with routine laboratory hematoxylin and eosin staining. Images were captured on an Olympus DP71 12.5-megapixel microscope mounted camera.

### 2.7. Cost and Length of Hospitalization

Using Kaiser State Health Facts [25], we estimated the average expense per inpatient day during the study period. The hospital length of stay (LOS) for inpatient encounters was determined for each case. Additionally, the number of hospital visits associated with symptoms or complications relating to their primary diagnosis of NCC was noted by reviewing the EMR's encounter numbers and visit reason.

### 2.8. Statistical Analysis

Frequencies and percentages were calculated for discrete variables. Mann–Whitney *U* test was applied to compare continuous variables and the chi-square or Fisher's exact test, as appropriate, was used for categorical variables. A comparison of medians was performed using a two-sample Wilcoxon rank-sum (Mann–Whitney) test. A *p* value of <0.05 was considered statistically significant for all tests. To compare our results to others [26], two groups (i.e., parenchymal and extraparenchymal) were compared by the univariate analysis. Data were analyzed with STATA 12.1 (StataCorp, College Station, Texas, USA). For incidence, we calculated the total number of new cases in Hispanics for the 12-year study divided by the total Hispanic population for Suffolk County; in addition, the incidence was calculated for those zip codes with more than five new cases.

### 2.9. Ethical Considerations

This study was reviewed and approved by the Institutional Review Board (IRB#836058) at Stony Brook University Hospital.

## 3. Results

### 3.1. Clinical-Epidemiological Features

Based on diagnostic criteria proposed by Del Brutto et al. 2001 [23], we identified 52 patients with NCC (*n* = 16 for 2005–2007; *n* = 8, 2008–2010; *n* = 3, 2011–2013; and *n* = 25, 2014–2016). Thirty-four (65%) patients were determined to have definitive NCC and 18 (35%) patients were identified as probable NCC. The median age was 35.0 years (range: 4–94), and 56% of patients in our study were male. Demographic data on race and ethnicity were available for all but one patient. Forty-eight (94%) patients were self-identified as ethnically Hispanic or Latino. The remaining three patients were identified as non-Hispanic white, Haitian, and South Asian, respectively. The record of the country of birth was available for 36 patients; the majority emigrated from Latin American countries (Guatemala *n* = 10, El Salvador *n* = 11, Honduras *n* = 4, Ecuador *n* = 7, Peru *n* = 1, Haiti *n* = 1, and Dominican Republic *n* = 1), and one patient was from the US. The average time since immigrating to the US was documented in 28 cases, and the median time from immigration into the US until a diagnosis of NCC was 9.0 years (range: 1 week to 30 years). Travel history was recorded for 21 patients; all were expatriates. Sixteen (30.7%) patients reported travel to an endemic country. The five patients who did not have a travel history to an endemic country had previously emigrated from an endemic country, on average 11 (range: 9–15) years prior to being diagnosed with NCC. We identified one adult, the US-born patient diagnosed with NCC who had known repeat travel to the Dominican Republic. Over half (53.8%) of the patients in this study presented initially with a chief complaint of headache, followed by nausea (15.3%), vomiting (13.4%) or a focal neurologic sign (13.4%). Twenty (38.5%) patients diagnosed with NCC initially presented to SBUH after the onset of seizures. Eight out of 23 patients tested were found to have *T. solium* antibodies in serum. Seven were positive by ELISA (ARUP Laboratory, Associated Regional and University Pathologists, Salt Lake City, UT), and one was positive by EITB (Centers for Disease Control and Prevention, Atlanta, GA). Three out of these eight patients also had detectable antibodies for NCC in cerebral spinal fluid by ELISA (ARUP Laboratory).

### 3.2. NCC Case Density by Location

**Figure 1:**
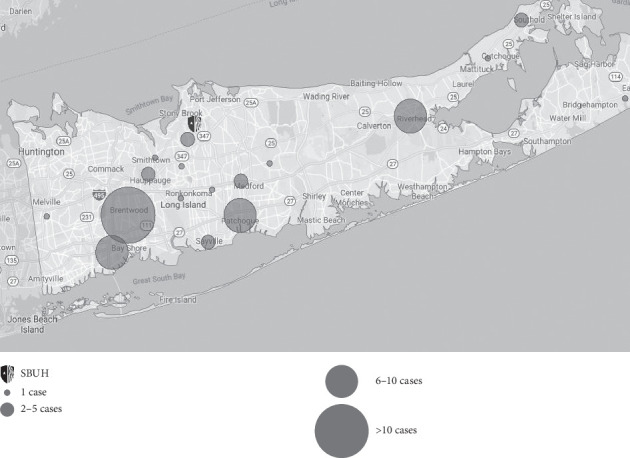
Map of Long Island, Suffolk Country, NY, with clusters of NCC cases documented from 2005 to 2016. Cases clustered by corresponding zip code.

The calculated incidence of NCC in our study was 1.6 cases per 100,000 people of Hispanic ethnicity. Forty-eight cases in our study occurred in Hispanics, and the average population in Suffolk County for Hispanics from 2010 to 2015 was approximately 248,695 people. Over 30% of patients in our study resided in zip codes where the Hispanic community accounts for approximately 65% of the local inhabitants. There are four zip codes with more than five reported cases during the study period: 11706 (*n* = 6), 11772 (*n* = 6), 11901 (*n* = 7), and 11717 (*n* = 11) (Figure 1). Based on the 2015 population data for these same zip codes, the incidences per 100,000 Hispanic residents are 0.74, 1.1, 1.9, and 1.49, respectively.

Twenty-five (48%) patients were diagnosed with NCC during 2014–2016, which was markedly higher compared to the other years evaluated in our study. The average age of patients during this period was 41 years (range: 15–94) and, on average, these patients immigrated 8.25 years prior to being diagnosed with NCC. The majority of these cases resided in the zip codes 11706 (*n* = 3), 11717 (*n* = 5), 11772 (*n* = 5), and 11901 (*n* = 5), which corresponded to areas with larger Hispanic communities.

### 3.3. Radiological Findings

Forty (76.9%) patients had one or more cysts in the parenchymal tissue on neuroimaging, 29 (55.7%) patients had cysts located in the subarachnoid space (convexity, sulcus, and basal cisterns), and 6 (11.5%) patients had ventricular cysts. Approximately 12 (23%) patients had an identifiable scolex reported on neuroimaging. Fifteen (28.8%) patients were found to have vasogenic edema associated with their cysts on neuroimaging, and 6 (11.5%) presented with hydrocephalus. No incidence of spinal cord cyst was noted in this study.

### 3.4. Radiological Classification

The clinical manifestations of NCC are a spectrum from asymptomatic to severely debilitating neurological disease, depending on the number, size, and location of the cysts and the degree of the host immune response. Classifying the disease is accomplished with neuroimaging reviewed by a neuroradiologist. Following the classification methodology described by Serpa et al. [26], patients with cysts in multiple locations were categorized by the least common site.

### 3.5. Parenchymal Cases

**Figure 2:**
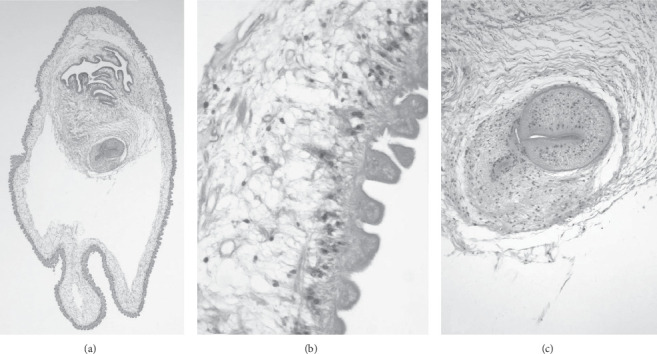
All three panels refer to the same specimen. (a) Low-power image of the larva of *T solium* surgically excised from the right frontal lobe (40x). (b) Detail of a region of the cyst wall reveals the characteristic undulating appearance of the microtrichia that form its outer layer, the middle cellular layer, and the inner loose reticular layer (600x). (c) Detail of the one sucker that is visible in the section (100x) (stain: hematoxylin and eosin).

Twenty-two (44%) patients diagnosed with NCC were found to have at least one parenchymal cyst. Ten (45%) patients with parenchymal lesions presented with seizures, twelve (54.5%) had headaches, and three (13.6%) presented with a focal neurologic sign. Of patients who were found to have parenchymal lesions, the most common stage identified (13 patients, 59%) was a calcified lesion. Nine (43%) patients were treated with antihelmintics and seven (32%) were treated with corticosteroids. Patients treated with antihelmintic medication received albendazole only. One patient (patient A) underwent resection of their cyst, as it was suspected to be a malignancy. Histopathology of the cyst revealed a necrotic parasite (Figure 2). Of the nine patients that had documented follow-up imaging, seven were noted to have improvement of their parenchymal lesions. The average hospital LOS for patients with parenchymal lesions was 7.2 days (range 1–31). These patients, on average, sought medical treatment for complications of NCC at least one time (average: 1.3 times) after their diagnosis.

### 3.6. Intraventricular Cases

**Figure 3:**
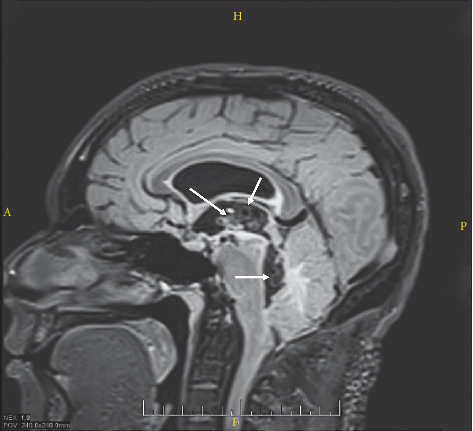
Multiple racemose cysts within the third and fourth ventricles (white arrows).

Six (12%) patients were classified as having intraventricular NCC based on neuroimaging (Figure 3). The most common presenting symptoms included headache (*n* = 4, 66.7%) and signs and symptoms of increased intracranial pressure (*n* = 3, 50%). One (16.7%) patient was found to have a focal neurological sign. None of the patients with intraventricular cysts presented with seizures. Radiographic evidence of hydrocephalus was present in four (66.7%) cases, and all required neurosurgical intervention with either a ventriculoperitoneal shunt (VPS) (*n* = 3, 50%) or an external ventricular drain (EVD) (*n* = 1, 16.67%). Five (83.3%) patients were treated with albendazole and corticosteroids. The treatment regimen was not documented for the sixth patient with intraventricular NCC. One patient was noted to have improvement of their intraventricular lesion after antihelmintic therapy. The average LOS for patients with intraventricular lesions was 14.8 days (range 1–49), and they sought medical treatment for complications of NCC on average 4.2 times during the study period (range 1–8).

### 3.7. Subarachnoid Cases

**Figure 4:**
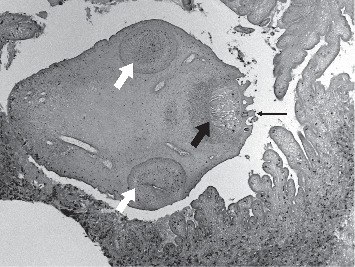
This photomicrograph shows a necrotic scolex of a larva of *T solium*, which is the large ovoid structure that is centrally located in this image (100x). Also present is the rostellum (thick, black arrow), hooks (thin, black arrow), and two ventral suckers (white arrows). The scolex is surrounded by fingers and fronds of the necrotic cyst wall that has mild neutrophilic inflammation (stain: hematoxylin and eosin).

Twenty-two (44%) patients had cysts in the subarachnoid spaces, which included subarachnoid space of the convexity (*n* = 8), sulcus of the convexity (*n* = 15), and basal cisterns of the subarachnoid space (*n* = 2). Headaches (*n* = 11, 50%) were the most common presenting complaint, followed by seizures (*n* = 9, 40.9%), signs and symptoms of increased intracranial pressure (*n* = 2, 9.0%), and focal neurologic deficits (*n* = 2, 9.0%). Hydrocephalus was seen on neuroimaging in one patient with subarachnoid lesions. Patients received treatment with steroids (*n* = 6, 27.3%), antihelmintics (*n* = 6, 27.3%), and surgery (*n* = 2, 9.1%). Albendazole was used alone in five patients, and one received treatment with both albendazole and praziquantel. Two patients required surgery during their hospitalization for NCC. One patient (patient B) underwent surgical resection by open craniotomy for diagnosis by histopathology. A parasite was visualized on histopathology (Figure 4). The average LOS for patients with subarachnoid lesions was 9.9 days (range 1–27), and they sought medical treatment for complications of NCC on average 1.5 times during the study period.

### 3.8. Differences between Parenchymal and Extraparenchymal NCC

We compared patients with parenchymal (*n* = 22) and extraparenchymal (intraventricular and/or subarachnoid) (*n* = 28) NCC. Patients with multiple lesions on neuroimaging were categorized based on the least common site. Two patients were excluded due to a lack of available neuroimaging in the EMR. In a univariate analysis (Table 2), patients with parenchymal disease were more likely to be younger, female, and present with seizures. Extraparenchymal cases were more likely to be male, older, and have longer hospitalizations. They were also more likely to be prescribed antihelmintics and steroids than parenchymal NCC cases. The most common presenting symptoms in both groups were headaches, and parenchymal cases were more likely to show radiographic evidence of improvement after treatment. The only significant difference between both groups was the average days of hospitalization, extraparenchymal (11.5 days) greater than the parenchyma group (7.2 days) (*p*=0.02).

### 3.9. Histopathological Findings

**Figure 5:**
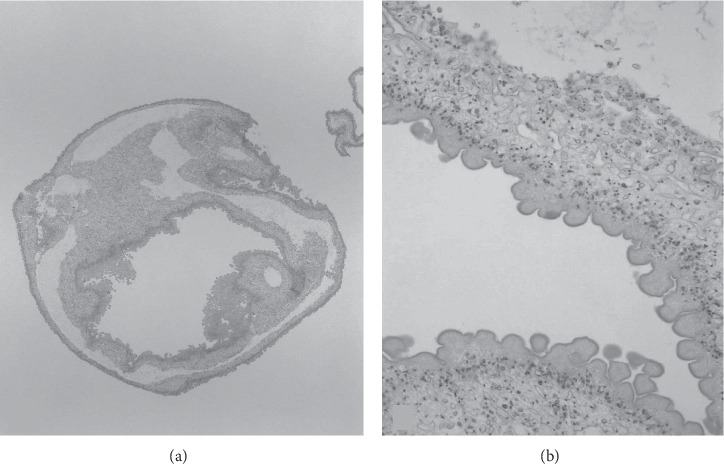
Both panels refer to same specimen.(a) This tissue, submitted as spherical lesions from the cervicomedullary junction, consists of one or more folded cyst walls of larvae of *T solium*. No scolex was found after examining multiple levels of the paraffin block (40x). (b) Detail of a region of the cyst wall shows the cuticular outer layer with multiple protrusions, the middle cellular layer, and the inner loose reticular layer that is characteristic of this organism (100x) (stain: hematoxylin and eosin).

Three patients underwent surgical resection of cysts by open craniotomy for the purpose of tissue diagnosis. A neuropathologist (RS) reviewed each case for our study, and a detailed description of the findings is provided for the figures. Patient A presented with a chief complaint of headache and a solitary parenchymal lesion with associated vasogenic edema on imaging was noted. The presumed diagnosis was a malignant tumor, but the histopathology revealed a cyst with a scolex (Figure 2). Patient B presented with a solitary subarachnoid lesion and chief complaint of nausea, vomiting, headache, and seizures. A lesion was excised from the medial left temporal lobe. On histopathology, a larval worm was seen (Figure 4). Patient C underwent resection of a cervicomedullary junction cyst for decompression of a Chiari type 1 malformation. A VPS was placed for concomitant hydrocephalus. A characteristic *T. solium* cyst wall lacking a scolex was described on histopathology (Figure 5).

### 3.10. Complications

Five patients in our study were VPS- or EVD-dependent due to complications from ventricular NCC infections and required frequent hospitalizations and neurosurgical interventions during the study period. One patient with a VPS did not have neuroimaging available for review, but imaging reports were available, which indicated the presence of racemose cysts and ventriculitis in addition to hydrocephalus. One patient had an EVD placed, during hospitalization for hydrocephalus, after resection of a subarachnoid cyst. Headache (*n* = 4, 80%), signs and symptoms of increased intracranial pressure (*n* = 3, 60%), and cranial nerve palsies (diplopia and sensorineural hearing loss) (*n* = 2, 40%) were the most common presenting symptoms for this subset of patients. The average LOS (20.25 days, range 2–49) for patients with a VPS was the longest of all NCC patients, and multiple hospitalizations (an average of 4 readmission during the study period) and surgeries were the result of frequent shunt or drain malfunction.

### 3.11. Treatment and Outcomes

The diagnosis and treatment of these patients predated the 2017 Clinical Practice Guidelines by the Infectious Diseases Society of America and the American Society of Tropical Medicine and Hygiene on the diagnosis and treatment of NCC [27]. Nineteen (44.2%) patients were treated with albendazole alone in our study, and two received a combination of albendazole and praziquantel. Nineteen (44.2%) patients also received corticosteroids, and seventeen of these patients received a combination of antihelmintics and corticosteroid. Eight patients required surgical intervention due to complications from their NCC infections, five of which had either a VPS or EVD placed (see complications section). Three patients had cysts resected (see histopathology section). One of the patients that had a cyst removed also had a VPS placed. All of them received antihelmintic therapy after cyst removal. No deaths due to NCC were recorded during our study period.

### 3.12. Estimated Cost of Hospitalization Due to NCC

We approximated the expense per inpatient day during the study period as 2,007 United States dollars (USD). This amount was determined from Kaiser State Health Facts and based on the estimated cost per inpatient day from 2005 (1,539 USD) to 2015 (2,475 USD) [25]. From 2005 to 2016, there were 74 hospital visits at SBUH due to symptoms or complications related to NCC. The average hospital LOS was 9.6 days (range: 1–49). Assuming that the daily cost of hospitalization is 2,007 USD, the total cost per patient visit to the hospital was $19,267 before factoring in the cost of neuroimaging, diagnostic tests, and surgical interventions. We estimate that the minimum accumulated cost of NCC hospitalizations at SBUH during the study period was over 1.4 million USD. Almost half (42.3%) of all patients' charts did not list a health insurance, including two of the VPS-dependent patients leading us to speculate that these patients were uninsured. Sixteen (30.7%) claimed a government health insurance, and 11 (21.1%) patients had commercial health insurance.

## 4. Discussion

**Figure 6:**
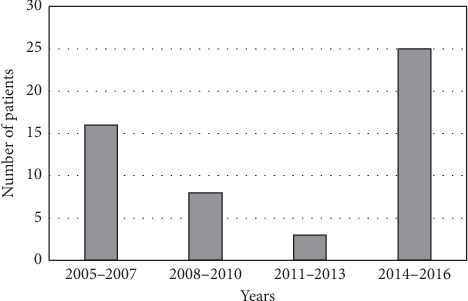
Graph depicting numbers of NCC cases diagnosed at Stony Brook University Hospital from 2005 to 2016.

We found 52 cases of NCC in a 12-year period at Stony Brook University Hospital. The vast majority (88%) were young, Hispanic adults who were residents primarily in two large Hispanic communities in Suffolk County, NY. The number of cases admitted to SBUH with a diagnosis of NCC has increased dramatically during the last three years of this study, which is likely due to the growth of the Hispanic community in Long Island, in addition to the widespread use of neuroimaging in emergency departments (Figure 6). Healthcare providers are becoming more aware of NCC in this region, although improved education on the diagnosis and management of NCC is still needed. This is especially true in community hospitals where an infectious diseases specialist may not be available.

In the US, the incidence of NCC is poorly understood, partly due to the transient nature of at-risk populations and the diagnostic challenges of NCC. The highest incidence in our study was 1.9 per 100,000 people in a region where there is a large Hispanic community, which was similar to others [9]. As the number of NCC cases continues to rise in the US, healthcare providers practicing in areas with large Hispanic communities should have a high index of clinical suspicion for NCC as part of their differential diagnosis in patients with adult-onset seizures or neurological symptoms. NCC is an increasing public health concern in the US, especially among the Hispanic community. The cost of this NTD to the US healthcare system is an estimated 908 million USD from 2003 to 2012, exceeding the cost of all other neglected tropical diseases combined [17]. Compared to other neurological diseases, such as epilepsy and multiple sclerosis, which annually costs the US 13.4 billion USD and 24.2 billion USD, respectively [28], NCC generates a comparable financial burden on the healthcare system. NCC is considered an NTD in the US due to poor access to healthcare by the communities most affected by this disease and underdiagnosis by healthcare providers who are unfamiliar with the management of this infection. Among immigrant populations, barriers to obtaining appropriate and timely healthcare include poverty, language, degree of acculturation, and fear of immigration policing agencies, among others [29, 30]. These factors were likely at play in the cases of NCC found in this study, as the majority of patients were prior residents of Latin American countries and, on average, were residents in the US for only four years. These findings were similar to other studies [31]. In addition, most of our patients did not have documented health insurance during their hospitalization. Whether these complications could have been avoided by earlier diagnosis or better healthcare access is an open question. This study brings awareness of NCC on Long Island, NY, and highlights the need for further education of healthcare providers practicing in areas with growing immigrant populations on NTDs, such as NCC. From this study, we hope to engender discussion among experts regarding future studies that will need to be conducted to answer whether or not serological or neuroimaging screening is beneficial and efficacious in diagnosing NCC earlier in hopes of preventing the severe consequences of this insidious infection.

## 5. Limitations

Limitations of this study include the retrospective nature and relatively small sample size. Data on patients' diagnoses, treatments, and outcomes are limited to data made available in the EMR. Our results are limited to patients at a tertiary academic medical center and may not apply to other populations. To the best of our knowledge, no prior studies on NCC in Long Island, NY, have been published.

## Figures and Tables

**Table 1:** Case diagnostic criteria for NCC [23].

Absolute criteria	(1) Histologic demonstration of the parasite in a biopsy of the central nervous system (CNS) tissue
	(2) Cystic lesion with scolex on imaging
	(3) Visualization of parasite by funduscopic examination

Major criteria	(1) Lesions highly suggestive of NCC on neuroimaging studies
	(2) Positive serum *T. solium* antibodies
	(3) Resolution of intracranial lesions after antiparasitic treatment
	(4) Spontaneous resolution of a small single lesion

Minor criteria	(1) Lesions compatible with NCC on neuroimaging studies
	(2) Clinical manifestations suggestive of NCC
	(3) Positive CSF enzyme-linked immunosorbent assay (ELISA) for *T. solium* antibodies or antigens
	(4) Cysticercosis outside of the CNS

Epidemiologic criteria	(1) Household contact with known *T. solium* infection
	(2) History of frequent travel to an endemic area

**Table 2:** Univariate analysis, parenchymal vs. extraparenchymal NCC cases 2005–2016.

	Parenchymal (*n* = 22), no. (%)	Extraparenchymal (*n* = 28), no. (%)	*p*
Age (years)^*∗*^	33.1 (range 4–94)	35.4 (range 15–93)	0.59
Males	9 (41)	19 (68)	0.08
Time from immigration to date of admission for NCC-related symptoms^*∗*^	10.3 (range 1 month–30 months)	8.5 (range 1 week–19 weeks)	0.21
Duration of hospitalization (days)^*∗*^	7.2 (range 1–31)	11.5 (range 1–49)	0.02
Patients readmitted for NCC-related symptoms	4/18 (22.2)	11/22 (50)	0.1
Seizures	10/22 (45)	9/26 (35)	0.55
Headaches	12/22 (54)	15/26 (56)	0.76
Use of surgery	1/22 (4.5)	6/28 (21.4)	0.11
Use of antihelmintics	9/21 (43)	11/22 (50)	0.76
Use of corticosteroids	7/22 (32)	11/22 (50)	0.35
Radiologic improvement on follow-up imaging	7/9 (78)	3/8 (37.5)	0.15

^*∗*^Mean and range are displayed.

## Data Availability

The medical record data used to support the findings of this study have not been made available. Although the data are de-identified, patients were not consented for this study due to its retrospective nature and, therefore, did not provide consent for public release of their individual health information. The authors share an ethical concern regarding unintended violations of patient privacy and legal concerns surrounding the immigration status of our patient population.
